# Extending pathways based on gene lists using InterPro domain signatures

**DOI:** 10.1186/1471-2105-9-3

**Published:** 2008-01-04

**Authors:** Florian Hahne, Alexander Mehrle, Dorit Arlt, Annemarie Poustka, Stefan Wiemann, Tim Beissbarth

**Affiliations:** 1 German Cancer Research Center, Molecular Genome Analysis, Im Neuenheimer Feld 580,69120 Heidelberg, Germany

## Abstract

**Background:**

High-throughput technologies like functional screens and gene expression analysis produce extended lists of candidate genes. Gene-Set Enrichment Analysis is a commonly used and well established technique to test for the statistically significant over-representation of particular pathways. A shortcoming of this method is however, that most genes that are investigated in the experiments have very sparse functional or pathway annotation and therefore cannot be the target of such an analysis. The approach presented here aims to assign lists of genes with limited annotation to previously described functional gene collections or pathways. This works by comparing InterPro domain signatures of the candidate gene lists with domain signatures of gene sets derived from known classifications, e.g. KEGG pathways.

**Results:**

In order to validate our approach, we designed a simulation study. Based on all pathways available in the KEGG database, we create test gene lists by randomly selecting pathway genes, removing these genes from the known pathways and adding variable amounts of noise in the form of genes not annotated to the pathway. We show that we can recover pathway memberships based on the simulated gene lists with high accuracy. We further demonstrate the applicability of our approach on a biological example.

**Conclusion:**

Results based on simulation and data analysis show that domain based pathway enrichment analysis is a very sensitive method to test for enrichment of pathways in sparsely annotated lists of genes. An R based software package *domainsignatures*, to routinely perform this analysis on the results of high-throughput screening, is available via Bioconductor.

## Background

Many high-throughput techniques such as DNA microarray analysis, siRNA screens or proteomic approaches result in extensive data. After careful statistical analysis the result of such an experiment is typically a list of candidate genes, relevant for certain biological processes, or an ordered gene list, sorted according to the significance in one or more biological processes [1]. The data analysis and interpretation of such lists provides a major bottleneck and a task for bioinformatics and systems biology. Many approaches have been published that assess the significant over-representation of biological functions or pathways as annotated in GO or pathway databases through gene set enrichment analysis [2-8].

However, for many of the screened genes there is hardly any functional annotation available. For example, the number of human genes annotated in the KEGG database [9] is only about 4,000. This contrasts the estimated number of putative protein coding genes which exceeds 23,000 (counted as the number of Entrez gene ids in the IPI-human database) [10,11]. Many approaches rely on automatically inferred functional annotations [12]. Such annotations can be assigned, for example, based on the protein sequences and predicted domains, as well as on protein interactions or co-expression. Structured vocabularies such as the Gene Ontologies (GO) comprise many such kinds of annotation [1].

Especially interesting and accessible is the functional annotation that is based on predicted protein domains, as there are already highly reliable prediction methods and databases available. Of the 23,000 genes in the IPI-human database, approximately 19,000 have at least one InterPro-domain assigned, of the 4,000 genes in the human-KEGG pathways, nearly all have at least one InterPro-domain. Together, these comprise approximately 3,000 distinct InterPro-domains [13]. Protein domains very often directly correspond to some of the core biological functions, such as e.g. DNA binding, kinase or phosphorylation activity, or otherwise to cellular localization. Therefore, predicted protein domains are often utilized to predict these annotations, for instance in the GO database. To our knowledge, however, protein-domain signatures have thus far not been used as a classifier to predict assignment of genes to pathways, which constitute the biological processes in a cell. Here we want to critically assess the utility of domain information to predict pathway membership and provide a tool to utilize this information. As we deem it unlikely to reliably predict pathway memberships for individual genes, our main aim is to find relevant pathways significantly enriched in lists of genes, in particular lists from high-throughput experiments. Pathways consist of a series of chemical reactions occurring within a cell. Of special importance for the functioning of biological systems are the signalling pathways, in which signals or cellular stimuli are transmitted mainly through protein interaction and phosphorylation events, often leading to altered gene expression within a cell. Known signalling pathways are collected within different pathway databases [14]. Pathways thus represent functional units for collecting genes or proteins. Crucial to gain hints of the function of genes is the mapping of these to one or more known pathways.

In many high-throughput experiments, we are facing the problem that we want to test for enrichment of pathways in gene lists, but the genes are often not annotated to pathways at all, or may even lack any form of functional annotation. We tried to answer this problem with the following idea: It should be possible to map a high fraction of the human genes to pathways via their protein domains. Hypothesis: Protein domains can be treated as functional elements of the proteins, the set of interpro-domains of the proteins of one pathway could serve as its functional fingerprint. In order to test the validity of this idea, we designed a simulation study based on the KEGG database and the protein domain assignment in the IPI database. Virtually removing genes from the KEGG pathways, we demonstrate that we can place these genes back into the respective pathway with high accuracy. We have further applied the method to a biological dataset from a high-throughput siRNA screen and demonstrate, that we are able to get biologicaly meaningful results.

## Results and Discussion

**Figure 1:**
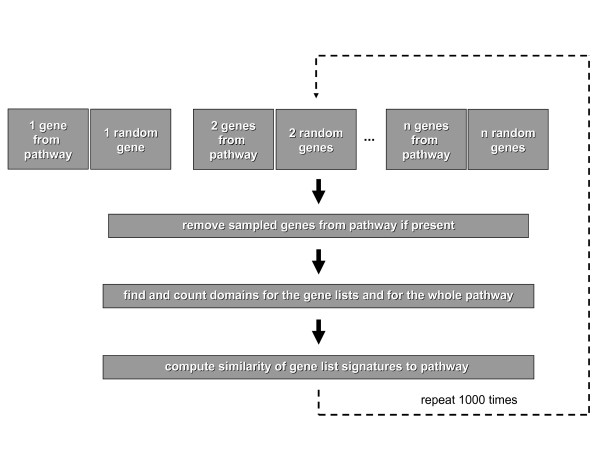
**Simulation work flow**. For the simulation study we sampled increasing numbers of genes from a given pathway and the same number of random genes. Genes that were present in both the pathway and the gene lists were removed from the pathway definition. Interpro domain signatures were constructed for both gene lists and for the pathway and measures of significance between gene lists and pathway were computed. This procedure was repeated 1,000 times and the resulting similarty measures were recorded.

We have developed a novel approach in order to test for the enrichment of pathways in arbitrary lists of genes. This approach is based on comparing the protein domain composition of the gene list of interest with the domain signature of the set of genes in each particular pathway. A more detailed description of this approach and a discussion of different measures of similarity, that can be utilized for this analysis, can be found in section *Similarity Measure*. We have tested the validity of this approach in a simulation study based on splitting the gene lists of known pathways from the KEGG database. We demonstate that the method works well for most known pathways even in the cases of noisy gene lists and pathway mixtures, at least for gene lists that contain 5 or more pathway genes. We suggest an approach to assess the significance of the resulting scores based on a resampling method in section *Assessment of significance *and test sensitivity and specificity in the assignment of pathway mixtures in section *Simulation of pathway mixtures*. Finally, we demonstrate the utility of our approach on a real biological dataset and discuss its interpretation. The method and simulation strategy is illustrated in Figure 1.

### Similarity Measure

There are different approaches to derive measures of similarity between the domain signatures of two distinct sets of genes. In the simplest case this is a binary distance based on the existence of a domain in a set of genes. We can create a binary vector reporting for each possible domain, whether it is part of any gene in the set or not and compute, for example, the Hamming distance between two vectors. However, this does not take into account the multiple appearances of domains in several genes of the set. In contrast, vectors of integer values reporting the number of appearance of all domains in the sets can be compared using simple Euclidean distance. During the development of the simulation strategy, we tested several different similarity measures, and showed that they led to comparable results. Here, we focus on a similarity measure that is more robust to deviations in the sizes of the sets, which is based on the chi-square statistic. For the two sets we count:

1. the number of intersecting domains for both sets,

2. the number of domains that are unique to set 1,

3. the number of domains that are unique to set 2,

4. the number of domains not part of any of the two sets.

By means of a binomial test, we compute on this contingency table the probability of over-representation of domains of set 1 in set 2 given all available domains. The negative log-transform of this probability can now be used as a measure for signature similarity. Note that higher values indicate a higher similarity between the two sets.

### Simulation Strategy

In order to assess whether InterPro domains can be used as a tool for functional classification we conducted a simulation experiment. We chose the pathways represented in the KEGG database as test environment, since this database is readily available. In principle, however, the method should be applicable to any kind of functional classification. Assuming, that the domain structure of either a single protein or, more likely, of a group of proteins with related function should be sufficient for functional classification (i.e. to assign a pathway membership), we sampled different numbers of genes that are known to be part of a given KEGG pathway. These genes were then removed from the pathway to construct somewhat smaller virtual pathways, which were used in the further test. In a subsequent step, we tried to recover the pathway membership of the collections of removed genes, based on the computed similarity of their domain signature to the domain signature derived from all remaining proteins of the respective pathway. To assess the significance of this score, the similarity measure of the sampled genes was compared to that of a control subset consisting of randomly chosen genes not specifically annotated to the respective pathway. We expect the latter to be less similar to the pathway signature.

### Simulating a single pathway

**Figure 2:**
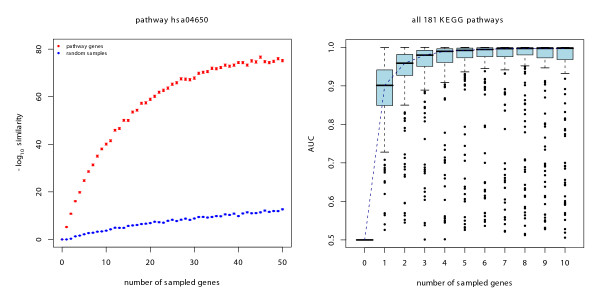
**Pathway classification**. Simulated classification of KEGG pathways based on a binomial similarity measure. a) Single pathway (hsa04650, natural killer cell mediated cytotoxicity). The red curve shows the similarity to the pathway for increasing numbers of sampled pathway genes. The blue curve shows the similarity to the pathway for increasing numbers of random genes. Each point comprises an average from 1000 independent samples and the sampling variance indicated by the error bars is negligible. b) Results for all 181 KEGG pathways. The boxplots show AUC values indicating the amount of separation between 1,000 random sampled gene lists and lists containing pathway genes for all pathways, again sampling increasing numbers of genes.

In an initial approach we chose a single non-metabolic pathway (hsa04650, natural killer cell mediated cytotoxicity) that comprises a high number of 126 annotated genes. For this pathway, we randomly sampled increasing numbers of member genes and computed the similarity measure to the remaining pathway genes. In parallel, the same increasing numbers of random genes that have not been specifically annotated to hsa04650 were sampled and the similarity was computed. For each number of genes, we repeated this procedure 1,000 times. In order to avoid over-fitting, the sampled pathway genes were removed from the data set used to train the classifier, hence they did not contribute to the global pathway signature they were compared against. The plot in Figure 2a shows the comparison of the computed similarities. While the domain signature of the random genes is not very similar to the pathway signature and stays more or less constant over different numbers of genes, we see a steady increase in similarity for increasing numbers of sampled pathway genes. Based on the signature of only two pathway genes we can already distinguish between the random sample and the pathway genes. The sampling variance indicated by the error bars is extremely low.

### Simulating all 181 pathways

In a second step, we performed a similar simulation for all 181 KEGG pathways, this time sampling sets containing from one up to ten pathway genes, each 1,000 times. To assess how well the pathway gene similarity and the random sample similarity separate, we computed ROC curves for each pathway and each sample number and, subsequently, the area under the curve (AUC) as a measure of separation. ROC curves are plots of the true positive rate against the false positive rate at different cutoff levels of a binary classifier. Thus, an AUC of 1 represents an ideal classification while an AUC of 0.5 is what can be achieved by random guessing. In the box plots in Figure 2b we plot the distribution of AUC values for all 181 pathways for the different numbers of sampled genes. For most of the pathways we achieve almost complete separation with only a small number of sampled genes. Already for two sampled genes, the bulk of the data lies above 0.9 indicating low misclassification rates. Sampling of five genes is sufficient to achieve complete separation for the majority of the 181 pathways. Still, some of the pathways can not be classified at all, or only very poorly. Detailed analysis of these pathways revealed that they often comprise only small numbers of proteins or that they belong to the class of metabolic pathways. In both cases, this result can be expectet: For small pathways we run into problems during the sampling process, because due to the removal of genes from the training set, we end up with sparse domain signatures based on a very small number of genes. Moreover, we do not expect a good performance of our method for many of the metabolic pathways since they are based on a functional classification that may only be weakly reflected in the composition of protein domains. Most of these domains are extremely unspecific, leading to a poor specificity of the overall domain signature. A detailed list of the performance of all 181 pathways can be found as Additional file 2.

### Simulating noisy data

**Figure 3:**
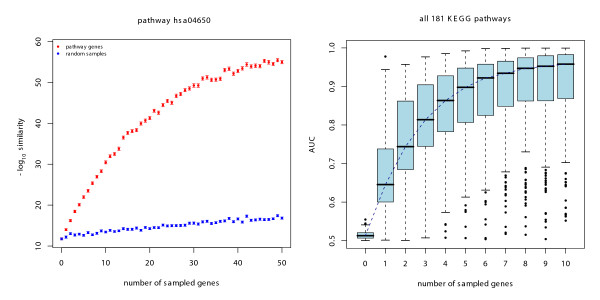
**Pathway classification with noisy data**. Simulated classification of KEGG pathways after addition of noise in the form of random genes that are not assigned to the respective pathways. The number of noise genes was fixed to 50. The similiarity measures and graphs are produced similar as for Figure 2. a) single pathway b) all 181 KEGG pathways.

In the previous sections, we based our simulations on ideal data, i.e. the gene list consisted purely of genes from a certain pathway. In a real application we will rather have to deal with gene lists that also contain 'noise genes' that do not belong to the respective pathway, or with a mixture of genes from several different pathways. In order to reflect this noise contribution, we extended our simulation strategy. Along with the pathway genes, we sampled a fixed number of 50 random genes, that are not annotated to the pathway of interest. Again, we computed similarity measures for theses sets and for random control sets and plotted the results (Figure 3). For the single pathway hsa04650 we can again clearly differentiate between the random gene sets and the gene set containing pathway genes. Absolute values of the similarity measures are lower compared to the 'clean' data set reflecting the noise contribution. The sample variance is again negligible. Upon application to all pathways the random gene sets can again be clearly separated from pathway gene sets based on only a small number of genes. For five genes in a list containing 50 noise genes we can almost perfectly separate the majority of all pathways.

### Assessment of significance

In order to apply our method to real biological data, one has to derive a measure of significance indicating that the similarity of the gene list to a given pathway is not merely by chance. Since we do not have a clear concept of probability distributions or statistical models of the highly complex pathway data, we applied a non-parametric sampling method to derive p-values for statistical significance. To this end, we sample for each pathway 10,000 random gene lists of the same size as the gene list analysed and compute their similarities to the pathway domain signature. The p-value is defined as the fraction of data points in this empirical distribution larger than the similarity measurement of the original gene list.

### Simulation of pathway mixtures

**Figure 4:**
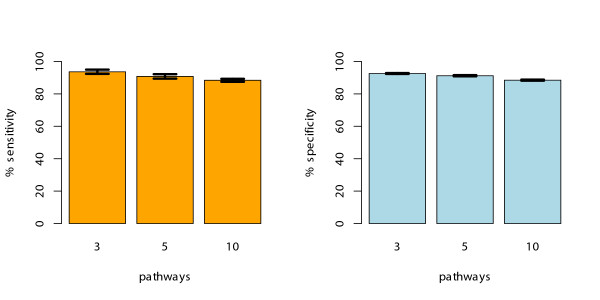
**Sensitivity and specificity**. Expected sensitivity (left) and specificity (right) of our method estimated through simulation of 100 sampled gene lists containing genes of varying numbers of KEGG pathways. 5 genes were sampled from each of 3, 5 or 10 different pathways, respectively, plus additional 50 random noise genes were added each time. The sensitivity and specificity was above 90% in all cases, the sampling variance indicated by the error bars is negligible.

Further, we studied in how far this approach is applicable for gene lists that contain genes from more then one pathway in addition to noise genes. We performed a simulation study and estimated the expected sensitivity versus specificity tradeoff of our method, meaning how many of these pathways we can recover and how many are lost. To this end we simulated putative gene lists, each containing 5 genes from either 3, 5 and 10 different randomly selected pathways, and added further 50 noise genes – so the total sizes of the simulated gene lists were 65, 75 and 100 genes, respectively. This procedure was repeated 100 times. Pathways were considered over-represented when the resulting *p*-values from our method were below 0.01. Subsequently, we computed sensitivity and specificity percentages from the total number of false positive and false negative pathway assignments (Figure 4). The 20 pathways showing domain signatures of very low predictive power (i.e. very small pathways with *AUC *< 0.7) as identified in the previous simulation were not regarded here. Based on these findings, our method is able to recover pathways with high accuracy both in terms of sensitivity and specificity of more than 90%. The slightly lower specificity values may be explained by the partially hierarchical structure of KEGG, with some of the pathways being part of other, more extended pathways (see Additional file 2 for estimates of similarities between individual KEGG pathways)

### Application to screening data

In order to demonstrate the applicability of our approach, we tested the method on a data set generated in the department in a genome wide high-throughput screen aiming to identify modulators of cell adhesion. This screen resulted in a list of approximately 1,000 candidate proteins. Application of our method revealed a significant similarity to the domain signature of the KEGG pathway hsa00531 for glycosaminoglycan degradation. Glycosaminoglycans (GAG) are linear polysaccharides built from disaccharidic units and they are covalently attached to core proteins, forming proteoglycans (PG) [15]. They provide mechanical links between the extracellular matrix and the cell surface, which influence signal transduction pathways and the cytoskeleton [16,17]. Proteins involved in glycosaminoglycan degradation are essential components in the regulation of GAG and PG functions. Down-regulation of genes involved in glycosaminoglycan degradation induces loss of cell adherence, and tumor stroma contains proteoglycans and glycosaminoglycans often in higher proportion than normal tissue [18]. As the biological role of the KEGG pathway hsa00531 fits to the phenotype which we screened with the assay for cell detachment, we conclude that our method is suitable for application to high-throughput data, revealing relevant proteins for potentially any cellular process. Furthermore, we could not detect a significant over-representation of hsa00531 in a classical hypergeometric test based on available KEGG annotations, indicating that our method adds additional information to gene lists compared to well established techniques.

## Conclusion

We have established a technique to use predicted protein domains in order to test for enrichment of pathways based on the domain signatures of the gene lists of interest. In a simulation study we show that our method can reliably predict the pathway membership of genes that were previously removed from a database of known pathways. To our knowledge, this approach is unique to assign pathway membership based on sequence information. This is important for prediction of biological functions and the basis for systems biological approaches. We demonstrate the applicability of our method in the functional analysis of high-throughput siRNA screening data and identify the enrichment of a biologically significant pathway in a poorly annotated gene list. This strategy will improve the field of gene set enrichment analysis and help to point out the biologically meaningful aspect of gene sets. The method is available as open source software in the Bioconductor package *domainsignatures *[19].

## Methods

### Databases

Gene to pathway mapping was done using information obtained as tab-delimited text files from the KEGG FTP-site [20], files hsa_pathway.list and hsa_ncbi-geneid.list. The files used in this analysis were downloaded in November 2006 and contain references to 181 pathways including 4,085 genes. The mapping of proteins to InterPro-domains was based on the information stored in the IPI database. The database files were downloaded from the IPI web site [21] in November 2006. This version of the IPI database contains 23,423 human genes (coding for at least one protein) and information for 3,968 genes which are also listed in the KEGG database. The Entrez GeneID [22] was used as the universal identifier. On average, each pathway contains 46,6 genes and 68.1 unique domains. On average each protein in the KEGG database has 3.5 unique domains. Each domain on average appears in 3.95 different pathways. Domains appear on average in 6.7% of all genes in a pathway and there are only very few domains that appear in the majority of genes of a pathway. Some further statistics on the distribution of domains in pathways and proteins are available in Additional file 2.

All databases were stored and queried via a local MS SQL server database.

### Implementation

All computations were done using the statistical programming language R in combination with Bioconductor tools. We provide a software package *domainsignatures *containing the means and source code to compute pathway enrichment in gene lists based on domain signatures (Additional file 1). The package uses the latest KEGG version based on the Bioconductor *KEGG *package and the domain annotation based on the *BiomaRt *package [23] and the annotation in the Ensembl [24] database.

### Detachment Assay

We used a biological dataset generated in the department as an example to test the utility of our method on the outcome of high-throuput experiments. The data is based on an RNAi screening experiment utilizing a cell detachment assay. This assay measured the number of floating cells and thus the loss of adherence after gene knock down in HEK293 cells transfected with siRNA pools. The siRNAs are available from Dharmacon (Lafayette). Transfections were performed using Lipofectamine (Invitrogen), and after 72 hours the culture medium was centrifuged to collect non-adherent cells. The cells were then fixed and counted by flow cytometry.

## Authors' contributions

AM and TB had the initial ideas for this study. TB and FH designed the concept of the methods and simulation studies. AM set up the local databases. FH and TB implemented the statistical methods and analysis. FH has run the simulations and applied the methods to datasets. The example screening dataset was provided by DA and SW. DA, AP and SW contributed in discussions. FH, AM, and TB wrote the manuscript. All authors read and approved the final manuscript.

## Supplementary Material

Additional File 2 Additional figures and statistics. Click here for file

Additional File 1 R package, source code of the method. Click here for file
