# Supplementary material for: Extending pathways based on gene lists using InterPro domain signatures
Source: BMC Bioinformatics. 2008 Jan 4;9:3. doi: 10.1186/1471-2105-9-3 (PMC2245903; doi:10.1186/1471-2105-9-3)
Supplement: Additional File 2 — Additional figures and statistics. [file 1471-2105-9-3-S2.pdf]

Extending pathways based on gene list signatures using InterPro domains - Appendix

Florian Hahne[†], Alexander Mehrle[†] , Dorit Arlt, Annemarie Poustka, Stefan Wiemann and Tim Beissbarth[†*]

German Cancer Research Center, Molecular Genome Analysis, Im Neuenheimer Feld 580,69120 Heidelberg, Germany
[†]Equal contributors

Email: Florian Hahne - f.hahne@dkfz.de; Alexander Mehrle - a.mehrle@dkfz.de; Dorit Arlt - d.arlt@dkfz.de; Annemarie Poustka - a.poustka@dkfz.de; Stefan Wiemann - s.wiemann@dkfz.de; Tim Beissbarth*- t.beissbarth@dkfz.de;

*Corresponding author

Additional File - Supplementary Material

Figure S1 — Domain signatures of all pathways
Figure S2 — Distribution of pathways, domains and genes
Figure S3 — Heatmap of pathway similarities
Table S1 — Separation Scores for all 181 pathways.

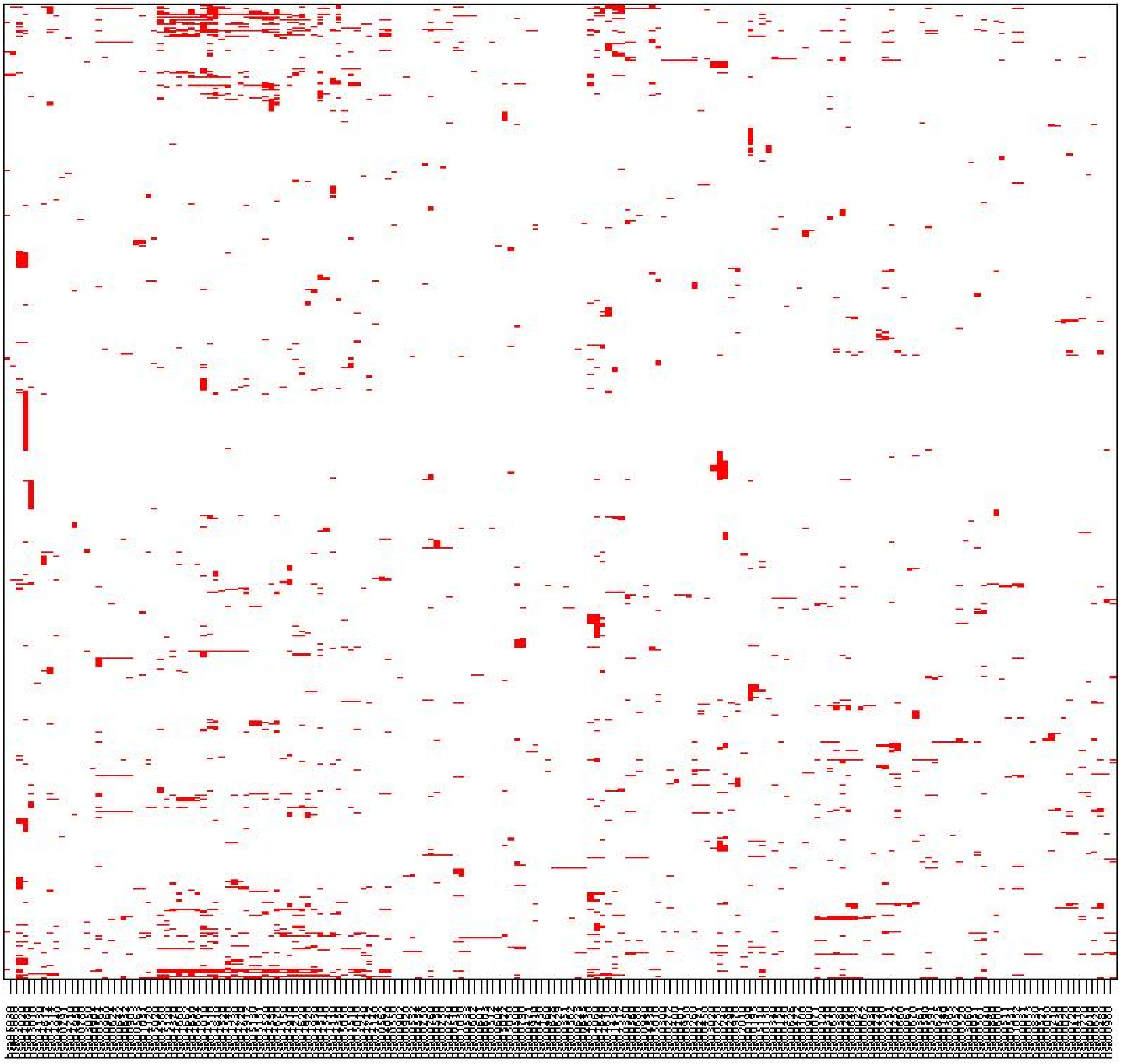

Figure 1: Plotted are pathways versus domains. Each domain that is contained one ore more times in a given pathway is indicated by red color. The matrix is extremely sparse.

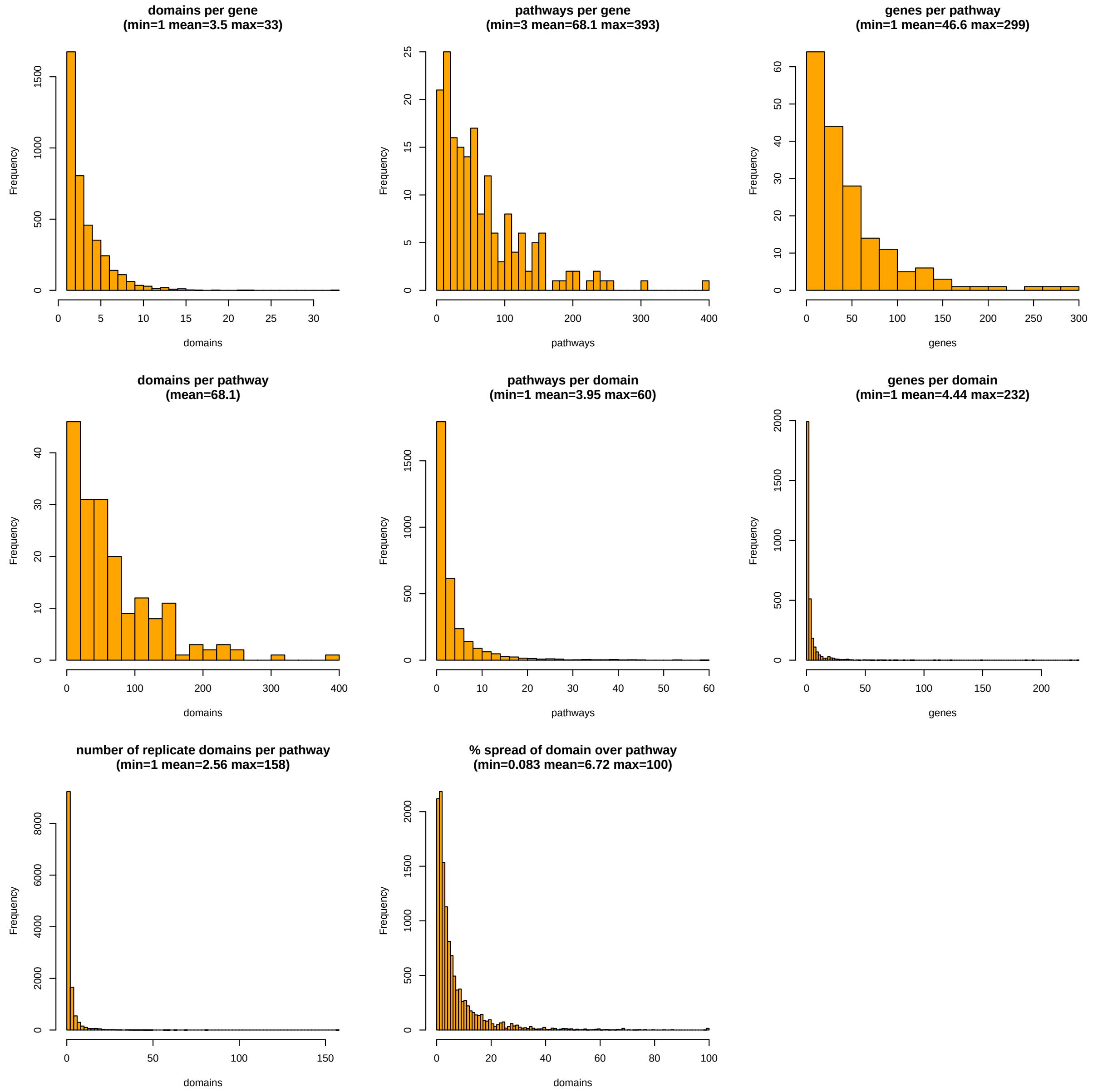

Figure 2: The distributions reveal that there are no pathway-specific domains that appear in every gene of a pathway.

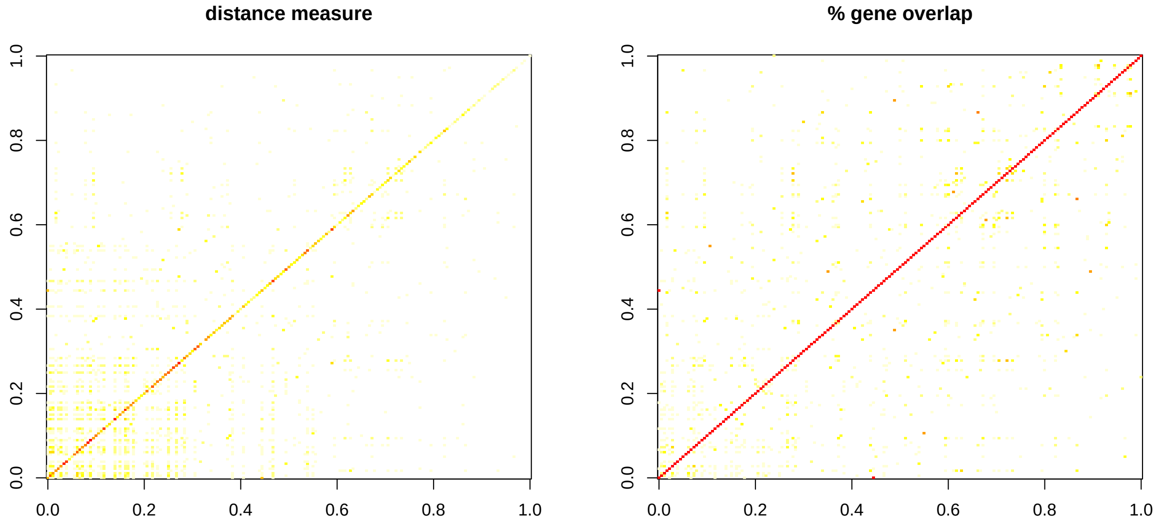

Figure 3: Similarity matrices between pathways. Left: using our similarity measure. Right: by relative gene overlap. Only very few of the 181 pathways show significant amounts of similarity.

Table 1: Separation Scores for all 181 pathways.

Pathway ID	Average AUC	Average AUC (noise)	Size	Description
hsa02010	0.95	0.91	171	ABC transporters - General
hsa00970	0.95	0.92	170	Aminoacyl-tRNA biosynthesis
hsa00071	0.95	0.91	170	Fatty acid metabolism
hsa00511	0.95	0.92	33	N-Glycan degradation
hsa00980	0.95	0.90	228	Metabolism of xenobiotics by cytochrome P450
hsa04130	0.95	0.90	91	SNARE interactions in vesicular transport
hsa04512	0.95	0.88	518	ECM-receptor interaction
hsa00534	0.95	0.90	21	Heparan sulfate biosynthesis
hsa04330	0.95	0.92	207	Notch signaling pathway
hsa04070	0.95	0.90	352	Phosphatidylinositol signaling system
hsa00710	0.95	0.90	69	Carbon fixation
hsa04610	0.95	0.90	384	Complement and coagulation cascades
hsa04710	0.95	0.90	80	Circadian rhythm
hsa04360	0.95	0.85	814	Axon guidance
hsa03050	0.95	0.92	86	Proteasome
hsa04664	0.95	0.84	387	Fc epsilon RI signaling pathway
hsa04514	0.95	0.83	682	Cell adhesion molecules (CAMs)
hsa01430	0.95	0.86	579	Cell Communication
hsa04720	0.95	0.85	324	Long-term potentiation
hsa00120	0.95	0.90	115	Bile acid biosynthesis
hsa00640	0.95	0.91	158	Propanoate metabolism
hsa04320	0.95	0.89	168	Dorso-ventral axis formation
hsa03030	0.95	0.91	72	DNA polymerase
hsa00562	0.95	0.90	213	Inositol phosphate metabolism
hsa00150	0.95	0.86	82	Androgen and estrogen metabolism
hsa00363	0.95	0.91	31	Bisphenol A degradation
hsa00053	0.95	0.91	29	Ascorbate and aldarate metabolism
hsa00361	0.95	0.90	56	gamma-Hexachlorocyclohexane degradation
hsa04912	0.95	0.82	434	GnRH signaling pathway
hsa00624	0.95	0.90	107	1- and 2-Methylnaphthalene degradation

Continued on next page

Pathway ID	Average AUC	Average AUC (noise)	Size	Description
hsa04662	0.95	0.85	312	B cell receptor signaling pathway
hsa00561	0.95	0.89	201	Glycerolipid metabolism
hsa00930	0.95	0.92	35	Caprolactam degradation
hsa00140	0.95	0.88	22	C21-Steroid hormone metabolism
hsa04730	0.95	0.86	339	Long-term depression
hsa00591	0.95	0.87	95	Linoleic acid metabolism
hsa04660	0.95	0.82	471	T cell receptor signaling pathway
hsa00602	0.95	0.88	38	Glycosphingolipid biosynthesis - neo-lactoseries
hsa04120	0.95	0.88	134	Ubiquitin mediated proteolysis
hsa00010	0.95	0.86	175	Glycolysis / Gluconeogenesis
hsa04612	0.95	0.83	312	Antigen processing and presentation
hsa04210	0.95	0.84	360	Apoptosis
hsa04740	0.94	0.82	131	Olfactory transduction
hsa00512	0.94	0.78	73	O-Glycan biosynthesis
hsa00480	0.94	0.88	155	Glutathione metabolism
hsa00020	0.94	0.85	119	Citrate cycle (TCA cycle)
hsa00791	0.94	0.91	24	Atrazine degradation
hsa04540	0.94	0.82	488	Gap junction
hsa04530	0.94	0.84	501	Tight junction
hsa04940	0.94	0.80	199	Type I diabetes mellitus
hsa04080	0.94	0.78	993	Neuroactive ligand-receptor interaction
hsa00620	0.94	0.89	152	Pyruvate metabolism
hsa04020	0.94	0.84	781	Calcium signaling pathway
hsa04110	0.94	0.85	367	Cell cycle
hsa03022	0.94	0.89	99	Basal transcription factors
hsa04950	0.94	0.90	101	Maturity onset diabetes of the young
hsa00590	0.94	0.85	167	Arachidonic acid metabolism
hsa04370	0.94	0.84	341	VEGF signaling pathway
hsa04520	0.94	0.81	385	Adherens junction
hsa04350	0.94	0.85	374	TGF-beta signaling pathway
hsa04650	0.94	0.79	537	Natural killer cell mediated cytotoxicity
hsa04310	0.94	0.83	590	Wnt signaling pathway
hsa04930	0.94	0.81	243	Type II diabetes mellitus
hsa00252	0.94	0.84	135	Alanine and aspartate metabolism
hsa04150	0.94	0.79	198	mTOR signaling pathway
hsa04340	0.94	0.79	200	Hedgehog signaling pathway
hsa04810	0.94	0.81	949	Regulation of actin cytoskeleton
hsa04670	0.94	0.84	570	Leukocyte transendothelial migration
hsa04510	0.94	0.81	1091	Focal adhesion
hsa03020	0.94	0.91	105	RNA polymerase
hsa00903	0.94	0.85	79	Limonene and pinene degradation
hsa00604	0.94	0.89	21	Glycosphingolipid biosynthesis - ganglioseries
hsa04630	0.94	0.83	586	Jak-STAT signaling pathway
hsa00272	0.94	0.90	49	Cysteine metabolism
hsa00440	0.94	0.91	32	Aminophosphonate metabolism
hsa00626	0.94	0.87	26	Naphthalene and anthracene degradation
hsa05130	0.94	0.82	219	Pathogenic Escherichia coli infection - EHEC
hsa00460	0.94	0.86	14	Cyanoamino acid metabolism
hsa03060	0.94	0.88	32	Protein export
hsa00280	0.94	0.88	205	Valine, leucine and isoleucine degradation
hsa00430	0.94	0.89	13	Taurine and hypotaurine metabolism
hsa00500	0.94	0.87	232	Starch and sucrose metabolism
hsa00310	0.94	0.86	156	Lysine degradation
hsa00603	0.94	0.90	23	Glycosphingolipid biosynthesis - globoseries
hsa00790	0.94	0.85	110	Folate biosynthesis
hsa05131	0.94	0.83	219	Pathogenic Escherichia coli infection - EPEC
hsa00360	0.94	0.83	87	Phenylalanine metabolism
hsa00410	0.94	0.86	82	beta-Alanine metabolism
hsa00193	0.93	0.84	84	-
hsa00564	0.93	0.84	203	Glycerophospholipid metabolism
hsa04010	0.93	0.75	1198	MAPK signaling pathway
hsa04060	0.93	0.77	878	Cytokine-cytokine receptor interaction

Continued on next page

Pathway ID	Average AUC	Average AUC (noise)	Size	Description
hsa04910	0.93	0.79	555	Insulin signaling pathway
hsa00030	0.93	0.87	83	Pentose phosphate pathway
hsa00740	0.93	0.91	35	Riboflavin metabolism
hsa00521	0.93	0.89	20	Streptomycin biosynthesis
hsa05120	0.93	0.75	246	Epithelial cell signaling in Helicobacter pylori infection
hsa04640	0.93	0.76	382	Hematopoietic cell lineage
hsa05050	0.93	0.77	90	Dentatorubropallidoluysian atrophy (DRPLA)
hsa01030	0.93	0.76	192	Glycan structures - biosynthesis 1
hsa00530	0.93	0.86	84	Aminosugars metabolism
hsa01031	0.93	0.80	108	Glycan structures - biosynthesis 2
hsa00650	0.93	0.84	122	Butanoate metabolism
hsa04742	0.93	0.79	131	Taste transduction
hsa00450	0.93	0.81	71	Selenoamino acid metabolism
hsa00720	0.93	0.90	43	Reductive carboxylate cycle (CO2 fixation)
hsa00532	0.93	0.90	27	Chondroitin sulfate biosynthesis
hsa00260	0.93	0.84	110	Glycine, serine and threonine metabolism
hsa00533	0.93	0.85	26	Keratan sulfate biosynthesis
hsa00052	0.93	0.86	80	Galactose metabolism
hsa00330	0.93	0.86	193	Arginine and proline metabolism
hsa00625	0.93	0.88	23	Tetrachloroethene degradation
hsa00340	0.93	0.86	88	Histidine metabolism
hsa00230	0.93	0.82	452	Purine metabolism
hsa00271	0.93	0.83	44	Methionine metabolism
hsa00251	0.93	0.83	140	Glutamate metabolism
hsa00240	0.93	0.81	306	Pyrimidine metabolism
hsa00061	0.93	0.83	44	Fatty acid biosynthesis
hsa00051	0.92	0.82	125	Fructose and mannose metabolism
hsa04140	0.92	0.65	78	Regulation of autophagy
hsa00630	0.92	0.89	34	Glyoxylate and dicarboxylate metabolism
hsa00350	0.92	0.78	167	Tyrosine metabolism
hsa03010	0.92	0.76	244	Ribosome
hsa00770	0.92	0.85	55	Pantothenate and CoA biosynthesis
hsa00400	0.92	0.86	43	Phenylalanine, tyrosine and tryptophan biosynthesis
hsa00290	0.92	0.86	45	Valine, leucine and isoleucine biosynthesis
hsa00600	0.92	0.80	69	Sphingolipid metabolism
hsa04920	0.92	0.71	307	Adipocytokine signaling pathway
hsa01032	0.92	0.74	71	Glycan structures - degradation
hsa04620	0.92	0.74	381	Toll-like receptor signaling pathway
hsa00601	0.92	0.84	14	Glycosphingolipid biosynthesis - lactoseries
hsa00062	0.91	0.86	25	Fatty acid elongation in mitochondria
hsa05040	0.91	0.81	117	Huntington's disease
hsa00642	0.91	0.74	43	Ethylbenzene degradation
hsa00920	0.91	0.88	24	Sulfur metabolism
hsa05110	0.91	0.75	133	Cholera - Infection
hsa00520	0.91	0.84	34	Nucleotide sugars metabolism
hsa01510	0.91	0.81	138	Neurodegenerative Disorders
hsa00380	0.91	0.74	232	Tryptophan metabolism
hsa00900	0.90	0.87	20	Terpenoid biosynthesis
hsa05030	0.90	0.81	55	Amyotrophic lateral sclerosis (ALS)
hsa00531	0.90	0.58	48	Glycosaminoglycan degradation
hsa00632	0.89	0.76	79	Benzoate degradation via CoA ligation
hsa00100	0.89	0.83	47	Biosynthesis of steroids
hsa00190	0.89	0.67	250	Oxidative phosphorylation
hsa00040	0.89	0.72	35	Pentose and glucuronate interconversions
hsa00670	0.89	0.73	55	One carbon pool by folate
hsa00860	0.89	0.69	73	Porphyrin and chlorophyll metabolism
hsa00510	0.88	0.69	69	N-Glycan biosynthesis
hsa00220	0.87	0.66	72	Urea cycle and metabolism of amino groups
hsa00750	0.86	0.77	29	Vitamin B6 metabolism
hsa00680	0.86	0.61	35	Methane metabolism
hsa00910	0.86	0.77	58	Nitrogen metabolism
hsa00072	0.85	0.83	29	Synthesis and degradation of ketone bodies

Continued on next page

Pathway ID	Average AUC	Average AUC (noise)	Size	Description
hsa00760	0.85	0.77	100	Nicotinate and nicotinamide metabolism
hsa00960	0.85	0.66	48	Alkaloid biosynthesis II
hsa05060	0.84	0.71	55	Prion disease
hsa00563	0.82	0.55	27	Glycosylphosphatidylinositol(GPI)-anchor biosynthesis
hsa00130	0.81	0.63	36	Ubiquinone biosynthesis
hsa00730	0.81	0.72	17	Thiamine metabolism
hsa00940	0.80	0.59	28	Phenylpropanoid biosynthesis
hsa00780	0.74	0.63	13	Biotin metabolism
hsa00362	0.74	0.70	10	Benzoate degradation via hydroxylation
hsa00830	0.73	0.72	7	Retinol metabolism
hsa00471	0.73	0.74	12	D-Glutamine and D-glutamate metabolism
hsa05010	0.72	0.60	78	Alzheimer's disease
hsa00904	0.71	0.77	10	Diterpenoid biosynthesis
hsa00950	0.69	0.68	20	Alkaloid biosynthesis I
hsa00401	0.66	0.68	16	Novobiocin biosynthesis
hsa00550	0.66	0.71	6	Peptidoglycan biosynthesis
hsa00472	0.66	0.71	5	D-Arginine and D-ornithine metabolism
hsa00031	0.64	0.64	5	Inositol metabolism
hsa00902	0.64	0.72	6	Monoterpenoid biosynthesis
hsa00623	0.63	0.81	12	2,4-Dichlorobenzoate degradation
hsa00351	0.62	0.60	7	1,1,1-Trichloro-2,2-bis(4-chlorophenyl)ethane (DDT) degradation
hsa00660	0.60	0.61	10	C5-Branched dibasic acid metabolism
hsa00628	0.59	0.63	4	Fluorene degradation
hsa00629	0.59	0.63	3	Carbazole degradation
hsa00643	0.59	0.71	13	Styrene degradation
hsa00300	0.57	0.68	11	Lysine biosynthesis
hsa05020	0.56	0.52	35	Parkinson's disease
